# Complete primary pachydermoperiostosis: A case report from Jordan and review of literature

**DOI:** 10.1002/ccr3.1971

**Published:** 2019-01-09

**Authors:** Bareqa I. Salah, Khalil I. Husari, Ala’ Hassouneh, Zaid Al‐Ali, Baeth Rawashdeh

**Affiliations:** ^1^ General Surgery Department Jordan University Hospital Amman Jordan; ^2^ Maxillofacial Surgery Department Jordan University Hospital Amman Jordan; ^3^ Special Surgery Department Jordan University Hospital Amman Jordan

**Keywords:** clubbing, cutis verticis gyrate, frontal rhytidectomy, hypertrophic osteoarthropathy, pachydermoperiostosis

## Abstract

Complete Primary Pachydermoperiostosis is a rare syndrome that presents with skin and skeletal manifestations. Though diagnosis can be made on the basis of the classic clinical and radiological features, it is often missed due to variable presentations. Therefore, it is important to know about this syndrome to reach correct diagnosis.

## 1 INTRODUCTION

Pachydermoperiostosis (PDP), also known as primary hypertrophic osteoarthropathy or Touraine‐Solente‐Gole syndrome, is a rare genetic disease with primary clinical features of pachydermia (thickening of skin) and periostosis (new bone formation). In 1935, three French dermatologists, Touraine et al, classified this condition as a familial disorder with three forms: *complete* (periostosis and pachyderma), *incomplete* (without pachyderma), and the *forme fruste* (pachydermia with minimal skeletal changes).^1^

The development of the disease usually starts during adolescence with gradual thickening of facial skin, which eventually resembles that of an elephant (pachyderm) and hence the name. The diagnosis should only be made when at least two out of a family history, hypertrophic skin changes, bone pain/radiographic changes, or clubbing are existing.^2^ The exact incidence is not known but estimated prevalence of the disease is 0.16%.^3^ We report a case of complete primary PDP that presented to Jordan University Hospital which is the second reported case of its kind in Jordan.^4^

## 2 CASE REPORT

An 18‐year‐old male Caucasian referred to plastic surgery clinic from dermatology department as a case of extensive skin folding on the forehead and depressed nasolabial fold. He also complained of bilateral knee joint pain and swelling. These symptoms were first noted at 16 years of age. No history of similar condition in family and consanguinity. No history of trauma and fractures.

**Figure 1:**
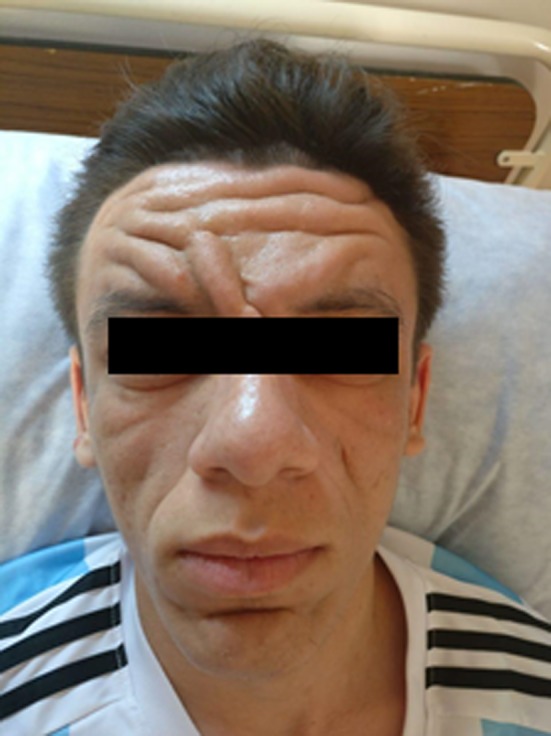
Preoperative photograph

**Figure 2:**
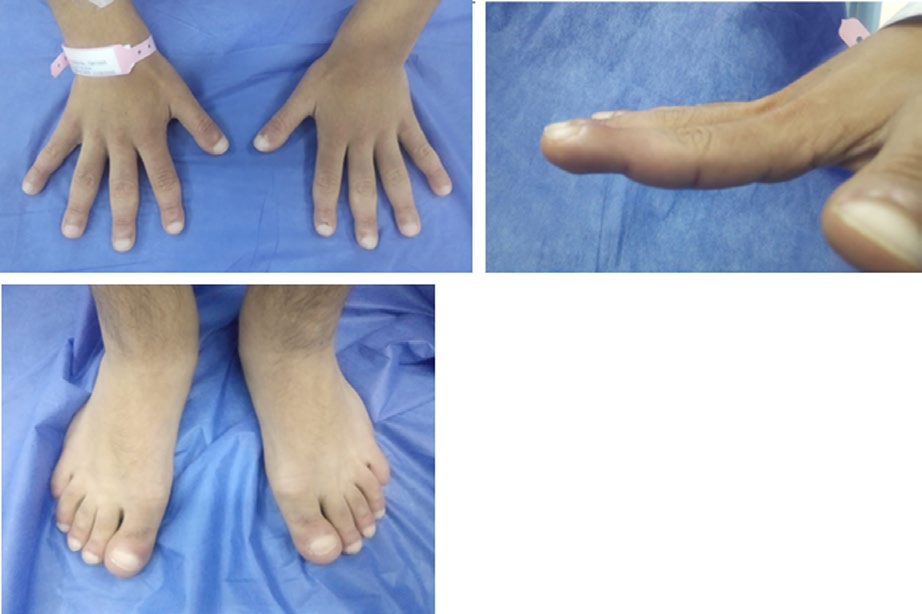
Digits clubbing

**Figure 3:**
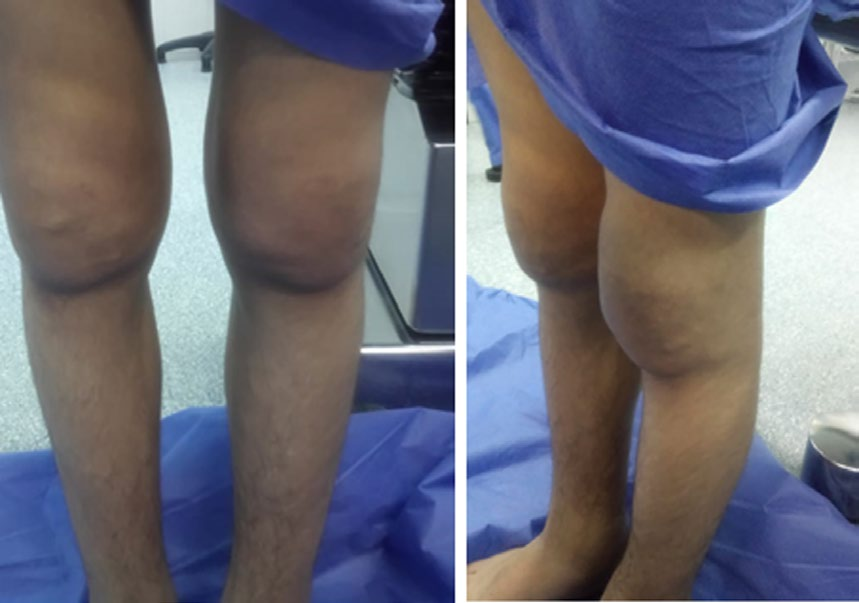
Swollen knee joints

On examination, he had pronounced folds in the area of forehead, between the eyes, in the nasolabial grooves and on the chin, furrowing on his forehead skin and first one inch of the scalp posterior to hairline, and bilateral partial ptosis (Figure 1). The development of the patient's skin folds was insidious and progressive. Clubbing of his fingers and toes (Figure 2) was noticed. Patient has swollen knee joints (Figure 3). Patient has profuse sweating and seborrhea in his axillae, hands, and feet.

Examination of the cardiovascular, respiratory, and gastrointestinal systems revealed no significant abnormalities.

Laboratory analysis showed a mild increase in ESR (18 mm/first h; normal < 15) and significant increase in C‐reactive protein (31.5 mg/L; normal < 5). The following parameters were normal: random blood sugar, serum calcium, growth hormone, and thyroid function tests.

**Figure 4:**
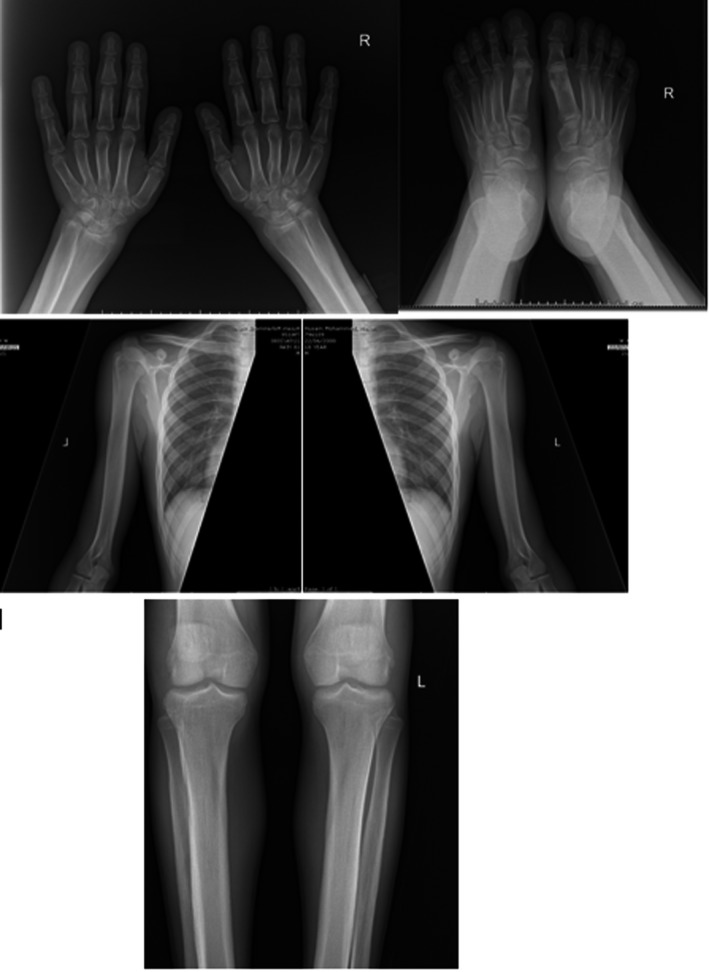
Hand X‐ray: periostosis, enlarged phalanges and metacarpals; Foot X‐ray: enlarged phalanges and metatarsals; Shoulder X‐ray: enlarged both clavicles and irregularity in the superiolateral borders of both scapulae indicating periostosis; and Knee X‐ray: symmetric shaggy subperiosteal bone formation with the involvement of epiphyseal regions, and speculated periostosis

Radiographic investigations were done to look for skeletal abnormalities. Plain X‐rays revealed thickening of the bone indicating increased bone formation, symmetric shaggy sub‐periosteal bone formation with the involvement of epiphyseal regions, acro‐osteolysis of the tufts of distal phalanges, irregularity in the superiolateral borders of both scapulae, and diffuse soft tissue thickening (Figure 4).

According to the data available from history, examination, and investigation, the patient was diagnosed with complete primary form of PDP.

**Figure 5:**
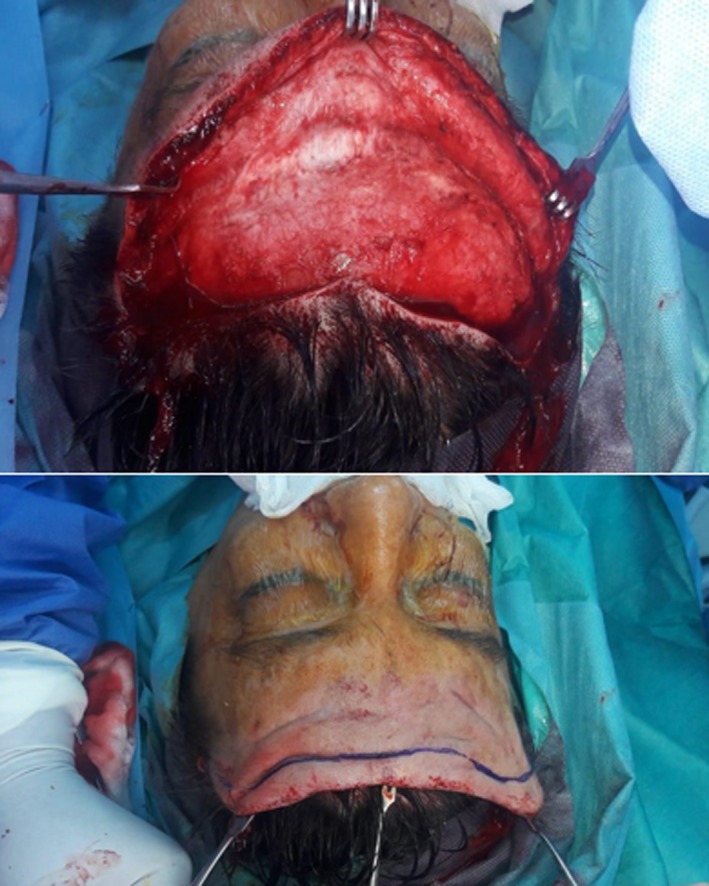
Frontal rhytidectomy

Two weeks after diagnosis, the patient underwent frontal rhytidectomy to remove excess skin in the forehead and to decrease the prominent nasolabial folds. A frontal rhytidectomy was performed through a brow lift incision, with dissection into forehead in the subgaleal and subfrontalis planes (Figure 5). Excision of 2 inches of excess forehead skin was achieved and sent for histopathological examination. Closure of skin with 3‐0 Nylon was done without tension.

Autologous dermal‐fat graft from the lower abdomen was placed in the nasolabial fold bilaterally through a small nasolabial incision, due to lack of enough fat available for liposuctioning and grafting. Postoperative recovery was unremarkable.

**Figure 6:**
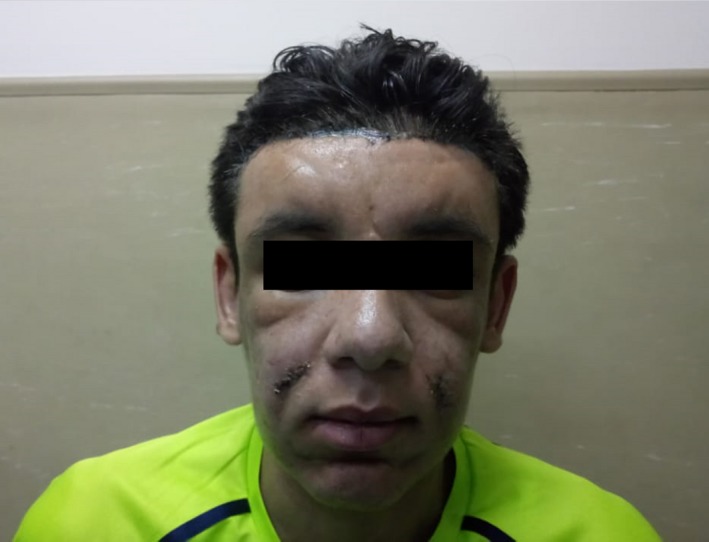
5 weeks after operation

Hyaluronic acid (1.6 mL of Juvederm^®^ filler) was injected in the forehead area 1 week after the operation, and Botulinum toxin type A (120 IU of Dysport^®^) was injected in the forehead muscles 4 weeks postoperatively (Figure 6).

He was satisfied with the operation cosmetic outcome. Future interventions might be needed with further progression of the disease.

**Figure 7:**
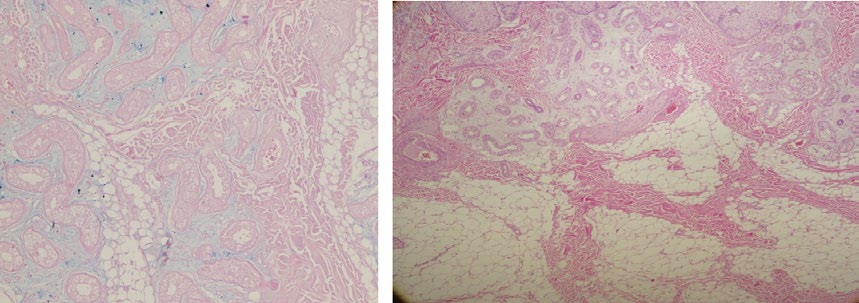
Left: Alcian blue special stain at power 100X, well‐controlled alcian blue and colloidal iron special stains highlight dermal per adnexal mucin deposition. Features of skin with deep dermal fibrosis, increased fibroblasts, and neuronal hyperplasia Right: H&E at power 40X, thickening of the dermis with fibrous bands extending into subcutaneous tissue surrounded by thick collagenous bundles with increased amount of ground substance

Microscopic evaluation of the excised skin revealed unremarkable epidermis and thickening of the dermis with fibrous bands extending into subcutaneous tissue surrounded by thick collagenous bundles with increased amount of ground substance. Well‐controlled alcian blue and colloidal iron special stains highlight dermal per adnexal mucin deposition. Features of skin with deep dermal fibrosis, increased fibroblasts, and neuronal hyperplasia were consistent with the clinical diagnosis of Pachydermoperiostosis (Figure 7).

## 3 DISCUSSION

Pachydermoperiostosis also known as Touraine‐Gole‐Solente syndrome was first described as “Hyperostosis of the entire skeleton” by Friedreich in 1868.^5^ In 1907, Unna suggested the term “cutis verticis gyrate” for thick, transversely folded skin of scalp and forehead.^6^ Three forms of this disease were described: *classic or complete form*, presented with skin and skeletal changes; *incomplete form*, with skeletal changes but no dermal findings; and *forme fruste* with dermal changes but no skeletal findings.^7^ When an affected person exhibits one or few features commonly associated with this syndrome, the diagnosis could be confirmed by thorough examination of other family members.^8^

Pachydermoperiostosis has a gradual onset. The disease usually starts at puberty, progresses variably for the next 5‐20 years, and then stays stable.^9^ It occurs more commonly in young male patients (M:F ratio of 7:1). In addition, males were found more severely affected. Major findings in this disease include pachydermia, periostosis, and finger clubbing. Other minor findings include hyperhidrosis, seborrhea, cutis verticis gyrate, blepharoptosis, joint effusion, arthralgia, column‐like legs, elephant feet, acne, and gastric ulcer.^10^

PDP makes 3%‐5% of cases of hypertrophic osteoarthropathy and should be distinguished from the *secondary form* before a diagnosis of PDP is established.^11^ The secondary form usually results from cardiopulmonary diseases (eg, bronchiectasis, cystic fibrosis, congenital heart diseases, and tuberculosis), hepatic diseases (eg, portal and biliary cirrhosis), gastrointestinal diseases (eg, inflammatory bowel disease and polyposis), and certain malignancies (eg, Hodgkin's disease, nasopharyngeal carcinoma, and chronic myeloid leukemia).

Clinically, in secondary form, the cutaneous findings (pachydermia, seborrhea, oiliness) are less frequent than primary PDP; the osteoarthropathy is more severe and painful, especially with congenital cyanotic heart disease.^12^ In secondary form due to neoplasia, only treatment of the underlying illness causes improvement of the associated symptoms.^13^

Other differential diagnoses include acromegaly, thyroid acropachy, van Buchem's disease (in which there is absence of clubbing and skin changes), psoriasis, and rheumatoid arthritis. Patients with forme fruste have to be differentiated from the rare hyperelasticity disorders such as Ehler‐Danlos syndrome, cutis laxa, Meretoga's syndrome, Marfan's syndrome, and pseudoxanthoma elasticum, which may cause forehead furrows.^14^

In up to 30% of the patients, PDP presents as a hereditary disease with autosomal dominance of variable penetrance.Although pathogenesis is currently unknown, an increased level of prostaglandin E2 which motivates the overexpression of the vascular endothelial growth factor has been proposed as a main factor. Due to high M:F ratio, X‐linked transmission and role of testosterone hormone have been suggested as other factors.^15^ Recently, hydroxyprostaglandin dehydrogenase (HPGD) and solute carrier organic anion transporter family member 2A1 (SLCO2A1) were described as pathogenic genes responsible for PDP. When germline SLCO2A1 mutations are detected, myelofibrosis, a life‐threatening complication, should be suspected and individual followed up periodically. Alcoholic consumption might be a contributing factor by alteration of prostaglandin metabolism.^16^ Unfortunately, genetic testing for our reported case was not available at our center.

Histopathological examination of skin samples taken from patients with PDP shows epidermal acanthosis and hyperkeratosis, different degrees of fibrosis and capillary ectasia of the dermis as well as sebaceous gland hypertrophy. Bone biopsy showed cortical hyperostosis and thickening of the periosteum with bands of partially hyalinized connective tissue in addition to vascular hyperplasia, with a reduction in trabecular bone. In cases with arthritis, the synovial membrane showed vascular congestion and stromal edema, lymphocytic and monocytic infiltration, and formation of solitary lymphatic follicles.^17^, ^18^ Radiographs of the hands and feet show joint space narrowing, swelling in the soft tissues, and acro‐osteolysis of the distal phalanges. There is also symmetrical periostosis that is more prominent in the distal lower limbs.^19^

The case presented here is a complete form of the syndrome, with the presence of most of the clinical characteristic and radiological findings. The patient had significant joint involvement and severe digital clubbing, and the presence of bony excrescences was detected in the X‐rays of his hands and feet. In this case, cutis verticis gyrate affected only his forehead and small area of the scalp. Furthermore, although this syndrome has a strong association with heredity, in this case, there was no report of relatives with similar characteristics.

After reviewing the literature for similar cases of Pachydermoperiostosis reported in our region (Arab world), we could find 17 reported cases distributed between Kuwait, Qatar, Tunisia, United Arab Emirates, and Jordan (Table 1).

**Table 1:** PDP cases reported from the Arab Region

No.	Author, Year	Age	Sex	Country	Clinical Presentation
1	Aburomman, 2014^4^	20	M	Jordan	Furrowing of forehead skin and scalp, digital clubbing, palmoplantar hyperhidrosis, and subperiosteal new bone formation
2	El Sawi, 1994^20^	22	M	Kuwait	Digital clubbing, coarse heavy features, and hyperhidrosis of the hands and feet
3	Al‐Emadi, 2006^21^	21	M	Qatar (Egyptian patient)	Greasy thick skin, deep nasolabial folds, furrowing of the scalp, partial ptosis of the eye, large tender joints, and severe digital clubbing
4	Al‐Emadi, 2006^21^	27	M	Qatar (Egyptian patient)	Similar presentation
5	Hamza M, 1977^22^	†	†	Tunisia	†
6	Haddad, 1977^23^	†	†	Tunisia	†
7	Rezgui‐Marhoul, 2005^24^	24	M	Tunisia	Distal polyarthralgia, thickening of face and scalp skin, greasy and shiny red face, and digital clubbing
8	Rezgui‐Marhoul, 2005^24^	58	M	Tunisia	Bilateral knee joint pain and swelling, limitation in the movement of hips, knee and elbow joints, digital clubbing
9	Ben Haj Slama, 2006^25^	†	†	Tunisia	Knee Joint swelling and pain
10	Akrout, 2012^26^	20	M	Tunisia	Morphologic abnormalities in face and extremities associated with skin changes
11‐16	Alaya Z, 2017^9^	18‐46	M	Tunisia	Thickening of skin of the head and distal extremities, deep folds and furrows of the forehead, periostosis of the long bones (6cases). Arthralgia (5 cases), polyarthritis (1 case), digital clubbing (5 cases), hyperhidrosis (4 cases), and seborrhea (2 cases)
17	Afify, 1986^27^	27	M	UAE	Thickening and folding of skin of face, spade‐like hands and feet with marked digital clubbing and hyperhidrosis

†, No data available.

According to our review of reported cases in the Arab world, we concluded that this disease is rare in our race. Genetics and environmental factors could play a role in its rarity. Nevertheless, its incidence could be probably under reported and undiagnosed cases could be present. Based on this, we recommend that further studies are needed to confirm the precise incidence and a comparison between different races has to be done. In addition, genetic testing for suspected patients and consideration of this disease as a differential diagnosis when facing such similar presentations are recommended.

No specific treatment exists; however, in most cases, PDP tends to stabilize over time.^28^ The therapeutic options for the control of symptoms consist of aspirin, nonsteroidal anti‐inflammatory drugs (NSAID), systemic corticosteroids, and colchicine.^29^ Da costa et al reported the use of infliximab in a patient with refractory arthritis.^30^ Plastic surgery is reserved for those patients with significant eyelid ptosis or those with severe aesthetic problems.^31^

## 4 CONCLUSION

Since PDP is a disease associated with stigmatization and a consequent reduction in the patient's quality of life, diagnosis of its various clinical forms and regular follow‐up by a team that includes a plastic surgeon, rheumatologist, and orthopedic are factors of ultimate importance. This report shows that, although rare, Pachydermoperiostosis should be considered in the differential diagnoses of similar presentation.

## CONFLICT OF INTEREST

None declared.

## AUTHOR CONTRIBUTION

BS: is a surgeon operator. KH: collected and analyzed the data. AH: wrote the manuscript. ZA‐A: designed the study. BR: is an assistant surgeon.
